# Comparative Transcriptomics Reveal Metabolic Rather than Genetic Control of Divergent Antioxidant Metabolism in the Primary Root Elongation Zone of Water-Stressed Cotton and Maize

**DOI:** 10.3390/antiox12020287

**Published:** 2023-01-27

**Authors:** Jian Kang, Sidharth Sen, Melvin J. Oliver, Robert E. Sharp

**Affiliations:** 1 Division of Plant Science and Technology, University of Missouri, Columbia, MO 65211, USA; 2 Interdisciplinary Plant Group, University of Missouri, Columbia, MO 65211, USA; 3 MU Institute for Data Science and Informatics, University of Missouri, Columbia, MO 65211, USA

**Keywords:** antioxidative metabolism, *Gossypium hirsutum*, orthologs, transcriptomics, roots, sulfur metabolism, water stress, *Zea mays*

## Abstract

Under water stress, the primary root elongation zones of cotton and maize exhibit both conserved and divergent metabolic responses, including variations in sulfur and antioxidant metabolism. To explore the relative importance of metabolic and genetic controls of these responses for each species, and the extent to which responses are mediated by similar gene expression networks within the framework of ortholog groups, comparative transcriptomics analyses were conducted under conditions of equivalent tissue water stress. Ortholog analysis revealed that 86% of the transcriptome response to water stress was phylogenetically unrelated between cotton and maize. Elevated transcript abundances for genes involved in abscisic acid (ABA) biosynthesis and signaling, as well as key enzymes that enable osmotic adjustment, were conserved between the species. In contrast, antioxidant responses, at least with regard to glutathione metabolism and anti-oxidative enzymes, did not exhibit such a transcript abundance adaptive signature. In particular, previously characterized differential responses of the glutathione and sulfur metabolic pathways between cotton and maize were not evident in the transcriptomic responses. The findings indicate that the antioxidant response in both species results from a metabolic acclimation to water stress, and thus represents an example of water stress-related metabolic plasticity.

## 1. Introduction

Drought is a major limitation to crop production globally [1], and understanding how growth and development are regulated in water-stressed plants is critical for efforts to develop crops with enhanced drought tolerance [2]. Root growth is generally less inhibited than shoot growth under water deficit conditions [3], and this differential response is an important adaptation that facilitates the maintenance of water uptake from resources deeper in the soil [4,5,6]. The ability for continued primary root elongation at low soil water potentials is vital for successful seedling establishment under water-limited conditions and has been documented in a wide range of species [7,8,9]. However, the genetic and metabolic mechanisms that determine the maintenance of primary root growth at low water potentials have not been investigated extensively in species other than maize [10] and, therefore, information on the generality of mechanisms across diverse species is very limited.

In the preceding paper [11], we explored this question by comparing the metabolic responses to water stress between the primary roots of cotton (*Gossypium hirsutum* L.) and maize (*Zea mays* L.). Cotton is a dicotyledonous perennial species that is cultivated commercially as an annual, in which the primary root forms the “tap” root as the foundation of the root system. In contrast, maize is a monocotyledonous plant in which the primary root functions primarily at the seedling stage. Experimental water-deficit conditions were defined that generated stable and equivalent tissue water potentials (approximately −1.6 MPa) in the primary root elongation zone of the two species. Under these conditions, root elongation rates were steady in both species and the elongation zones showed similar responses of cell proliferation and elongation. This characterization provided the foundation for a direct comparison of the biochemical and molecular responses to water stress in the root elongation zones of the two crops. Using a combination of global untargeted metabolite profiling and standard biochemical assays, the results revealed both conserved and species-specific metabolic regulatory mechanisms. The key differences between the species were in how antioxidative and sulfur metabolism responded to the equivalent tissue water stress [11]. In particular, cotton and maize showed contrasting glutathione responses as stress duration progressed, with glutathione levels declining in cotton but remaining elevated in maize. Despite the lesser response of glutathione in the cotton root, hydrogen peroxide (H_2_O_2_) levels were lower than in maize, in association with a more robust enzymatic antioxidant defense involving inherently higher catalase and superoxide dismutase activities and a stress-induced increase in ascorbate peroxidase activity that was not seen in maize.

What remains unclear from the previous study, however, is how these metabolic changes in response to water stress are mediated. Are the changes in metabolite abundance controlled by the regulation of the associated metabolic pathways in each species, or are there underlying genetic controls of the response(s)? If the root elongation zone responses are under genetic control in both cotton and maize, could this be mediated by similar gene expression patterns in both species? Understanding how these two crops control the metabolic responses underlying root growth maintenance at low water potentials is important for determining how the responses can be manipulated for crop improvement. In this report, we addressed these open questions by investigating differentially accumulated transcripts (DATs) in the primary root elongation zones of equivalently water-stressed cotton and maize seedlings, utilizing the comparative culture system previously developed [11]. The data generated allowed for the transcriptomic assessment of the relative importance of metabolic and genetic controls for each species and a direct comparison of the transcript abundance responses between cotton and maize roots within the framework of ortholog groups.

## 2. Materials and Methods

### 2.1. Plant Materials and Growth Conditions

**Figure 1:**
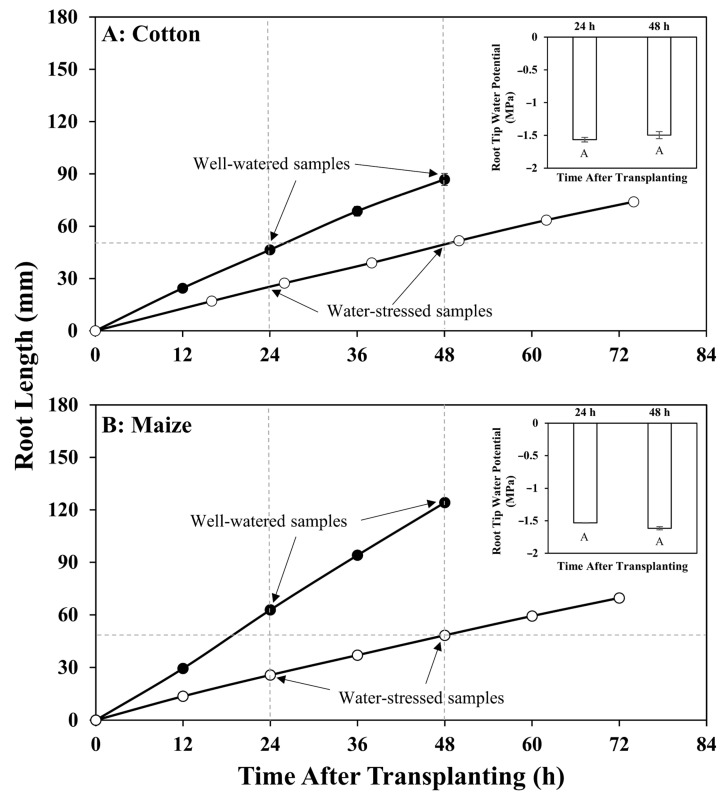
Increase in primary root length for (**A**) cotton and (**B**) maize seedlings with time after transplanting to well-watered or water-stressed conditions. Data points indicate times of root length measurement; dashed lines and arrows indicate the times (24 h and 48 h) at which root samples were collected for transcriptomics analyses. Insets show that the low water potential treatments (vermiculite water potentials: cotton, −1.0 MPa; maize, −1.6 MPa) generated equivalent tissue water potentials of approximately −1.6 MPa in the root elongation zone of both species at both sampling times. Data are reproduced with modification from Kang et al. [11], with permission.

Cotton (cv. AU90810) and maize (cv. FR697) seedlings were grown using a vermiculite culture system as previously described [7,11], in which the imposed water deficit treatments generated equivalent water potentials in the primary root elongation zone of the two species, indicating similar levels of tissue water stress. Seeds of cotton were produced in Prattville, AL, USA, and were provided by the Regional Breeders Testing Network, Cotton Inc. (Cary, NC, USA). Seeds of maize were produced at the University of Missouri in Columbia, MO, USA by self-pollination of plants from stocks originally obtained from Illinois Foundation Seeds Inc. (Tolono, IL, USA). These genotypes were chosen for both the preceding metabolic analysis and the present study because they are relatively water stress-tolerant in terms of primary root elongation [11,12]. Briefly, when the primary roots were 5–15 mm in length, seedlings were transplanted against the inner face of Plexiglas boxes containing vermiculite media (no. 2A, Therm-O-Rock East Inc., New Eagle, PA, USA) at water potentials of −0.02 MPa (well-watered control treatment), −1.0 MPa (cotton) or −1.6 MPa (maize) (water stress treatments). The different vermiculite water potentials were achieved by thorough mixing with differing volumes of 1 mM CaSO_4_ and were measured using isopiestic thermocouple psychrometry [13]. Under these defined water-deficit conditions, the primary root elongation zone of both cotton and maize exhibited steady water potentials of approximately −1.6 MPa at 24 h from transplanting (Figure 1). It should be noted that the cotton seedlings were not grown at the same vermiculite water potential as used for maize (−1.6 MPa) because, under this condition, the cotton root tip water potential was significantly lower than in maize, indicating a relatively greater hydraulic resistance to water uptake into the cotton root elongation zone [11].

Seedlings were grown at 29 °C in the dark and at near-saturation humidity to minimize further drying of the vermiculite until harvest at 24 h and 48 h after transplanting (Figure 1). Primary root tips encompassing the whole elongation zone of cotton (12 mm for well-watered, 6 mm for water-stressed) and maize (12 mm for well-watered, 7 mm for water-stressed), as defined by analysis of cell length profiles [11], were excised, immediately frozen in liquid nitrogen, and combined to generate samples of 50–100 mg fresh weight. Three replicate samples from independent experiments were collected for each treatment. Transplanting and harvesting were conducted using a green ‘safe’ light [14].

### 2.2. RNA Extraction and Library Construction

Total RNA was extracted from the root tip samples using the RNeasy^®^ Plant Mini Kit (QIAGEN, Redwood City, CA, USA). The RNA quality and quantity in each sample were assessed using a Bioanalyzer^®^ system (Agilent, Santa Clara, CA, USA) and Qubit^®^ Fluorometer (Invitrogen, Thermo Fisher Scientific, Inc., Waltham, MA, USA). Extracted RNA samples were diluted with nuclease-free water to a final volume of 50 µL for cDNA synthesis and the construction of strand-specific RNA-seq libraries using the NEBNext^®^ Ultra™ II RNA Library Prep Kit (Illumina^®^, San Diego, CA, USA). mRNA was isolated with Oligo-dT Beads d(T)25 (QIAGEN, Redwood City, CA, USA) and 10 µL aliquots were primed in NEBNext First Strand Synthesis Reaction Buffer with Random Primer Mix by incubation at 94 °C. The first DNA strand was synthesized by adding 10 µL of primed RNA to 10 µL of NEBNext First Strand cDNA Synthesis Reaction Mix containing 2 µL of NEBNext First Strand Synthesis Enzyme Mix and 8 µL of nuclease-free water. Subsequently, 60 µL of NEBNext Second Strand Synthesis Reaction Mix containing 8 µL of NEBNext Second Strand Synthesis Reaction Buffer, 4 µL of NEBNext Second Strand Synthesis Enzyme Mix and 48 µL of nuclease-free water was added to 20 µL of the first-strand product. The synthesized double-strand cDNA was purified, end-repaired and ligated to the NEBNext Adaptor. The cDNA samples were mixed with NEBNext Ultra II Q5 Master Mix containing Index Primers (Forward: 5′-CAAGCAGAAGACGGCATACGAGAT-3′; Reverse: 5′-AATGATACGGCGACCACCGAGATCTACAC-3′) and amplified by PCR. A total of 45 µL of NEBNext Sample Purification Beads was added to the PCR-enriched cDNA libraries for purification. The libraries were eluted in 23 µL of 0.1× TE Buffer, and stored at −20 °C. One µL of purified DNA was loaded onto a DNA High Sensitivity Chip and analyzed using the Bioanalyzer^®^ system (Agilent, Santa Clara, CA, USA) to determine if the libraries were composed of fragments within the expected size range of approximately 300 bp and of sufficient quality for sequencing.

### 2.3. RNA-seq and Differentially Accumulated Transcripts

Samples were shipped to the University of Missouri DNA Core Facility (Columbia, MO, USA) (cotton) or GENEWIZ Inc. (South Plainfield, NJ, USA) (maize) for sequencing. Cotton and maize samples were sequenced using single-end and paired-end modes of Illumina^®^ (San Diego, CA, USA) sequencing equipment, respectively. RNA-seq data are accessible in the GEO database (Accession number: GSE221296). The quality of the raw sequencing data was determined using FASTQC (v0.11.5) (https://www.bioinformatics.babraham.ac.uk/projects/fastqc/; accessed on 4 December 2022). The data were processed with Hisat2 (v1.0) [15,16] for the alignment of sequence reads to the cotton (NAU-NBT v1.1) and maize (AGPv4) genomes with a specific assembly for cv. FR697 [17] and to generate output files for Htseq-count (v0.6.1), to allow for the assessment of numbers of aligned reads that overlap the exon of each gene [18]. Read counts from the cotton and maize datasets were normalized with the Trimmed Mean of M-values (TMM) method. Using normalized read counts, edgeR (v3.14.0) was used to identify transcripts that changed significantly in abundance between treatments via an associated statistical algorithm [19]. All software packages were obtained from the CyVerse platform (www.cyverse.org; accessed on 4 December 2022). The final output established a database of normalized read counts for the transcripts of each gene, which was subsequently organized to produce lists of significant DATs from comparisons between water-stressed samples with well-watered controls within each species, negating the need to adjust for differences in sequencing protocols. The significant DATs for each species were used as candidates in the ortholog analysis for cross species comparison. Details of the specific comparisons are provided in the Section 3. The pipeline for DAT analysis is shown in Figure S1.

### 2.4. GO Term and KEGG Pathway Analyses

DATs in each species were analyzed using AgriGOv2 (http://systemsbiology.cau.edu.cn/agriGOv2/; accessed on 4 November 2022) [20] to determine significantly enriched GO terms under water stress conditions. To determine which pathways were highly involved in the responses to water stress for each species, DATs were mapped to pathways in the KEGG database (https://www.kegg.jp/kegg/pathway.html; accessed on 6 November 2022) [21].

### 2.5. Ortholog Analysis

The ortholog analysis was accomplished using two strategies to robustly identify orthologs between cotton and maize. Firstly, the OMA browser (https://omabrowser.org/; accessed on 21 August 2022), an online database that provides listed orthologs between plant species, was used to generate matched orthologs between the cotton and maize genomes as described by Altenhoff et al. [22]. Secondly, OrthoFinder [23] was used to predict ortholog groups and generate matched orthologs between the two species. As inputs for Orthofinder, proteomes derived from the phylogenetic tree of plants and available on Ensembl for monocot plants (including maize), Eurosids II (including cotton), and Asterids, encompassing 29 species in total, were used. Consensus ortholog groups were generated from the intersection of the two sources. Genes that were shown to be orthologs by both methods were considered as high confidence orthologs. Orthologs that were only supported by one source were considered to be low confidence orthologs.

### 2.6. Statistical Analysis

DATs were considered significant if exhibiting a log2-fold change in transcript abundance ratios between treatments at a significance level of FDR adjusted *p*-value of ≤ 0.05 with cutoffs of log2-FC ≤ −1 or ≥ 1. GO term enrichment results were filtered based on a FDR adjusted *p*-value of ≤ 0.05 and presented as −log10 (FDR adjusted *p*-value).

## 3. Results and Discussion

### 3.1. Overview of Differentially Accumulated Transcripts

The preceding study [11] centered on a non-targeted metabolite profiling strategy to gain a comparative assessment of the metabolic state of the primary root elongation zone in cotton and maize seedlings in response to equivalent tissue water stress conditions. The results revealed both conserved and species-specific metabolic regulatory mechanisms. Here, we utilized the same experimental system for a transcriptomic determination of the relative importance of metabolic and genetic controls of the stress-induced responses.

DATs were determined by comparing samples from the root elongation zone of water-stressed roots with well-watered controls. The primary comparison was conducted at 48 h after water stress imposition, at the same time as the previous metabolic assessment. At this time point, the water-stressed roots of both species had similar lengths and elongation rates (approximately 1.0 mm h^−1^) and exhibited stable root tip water potentials of approximately −1.6 MPa (Figure 1). As the water-stressed roots elongated more slowly than the well-watered roots, two well-watered control samples were utilized: a developmental control at 24 h (approximately the same root length as in the water stress treatment), and a temporal control at 48 h (same age as in the water stress treatment). DATs were defined as transcripts that significantly changed in the same direction (increased or decreased) in the water-stressed roots compared with both controls. Therefore, only changes associated with water stress and not with modifications of root development were included. The well-watered 24 h samples also served as a temporal control for water-stressed samples collected 24 h after stress imposition. This secondary comparison was used to assess whether the transcriptomic changes at 48 h reflected a steady stress response. A well-watered developmental control for the 24 h water-stressed samples was not collected.

**Figure 2:**
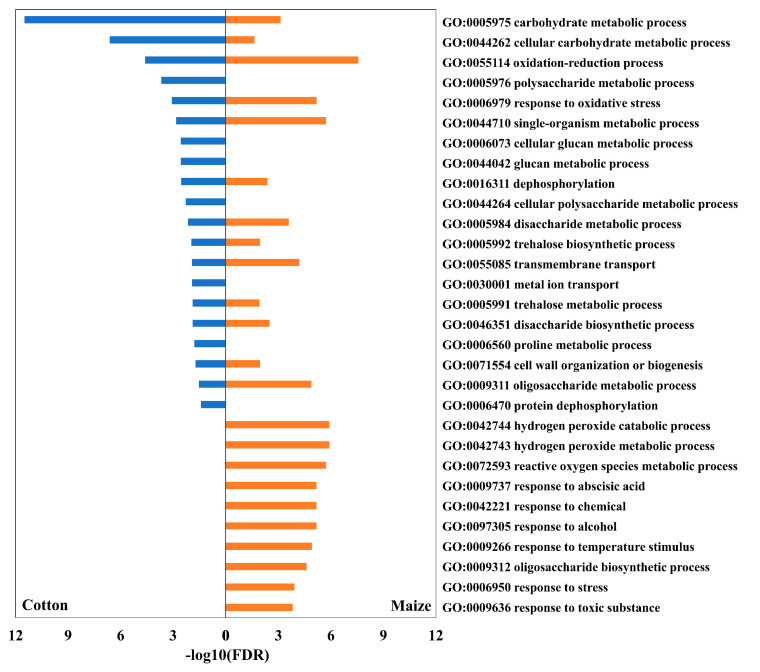
Biological process terms from GO enrichment analysis in the cotton and maize primary root elongation zones at 48 h after transplanting to the water stress treatment. All significant terms in cotton, with their corresponding values in maize, plus 10 additional terms with the highest significances in maize but not represented in cotton, are shown. Cotton: left panel (blue bars); maize, right panel (orange bars).

**Figure 3:**
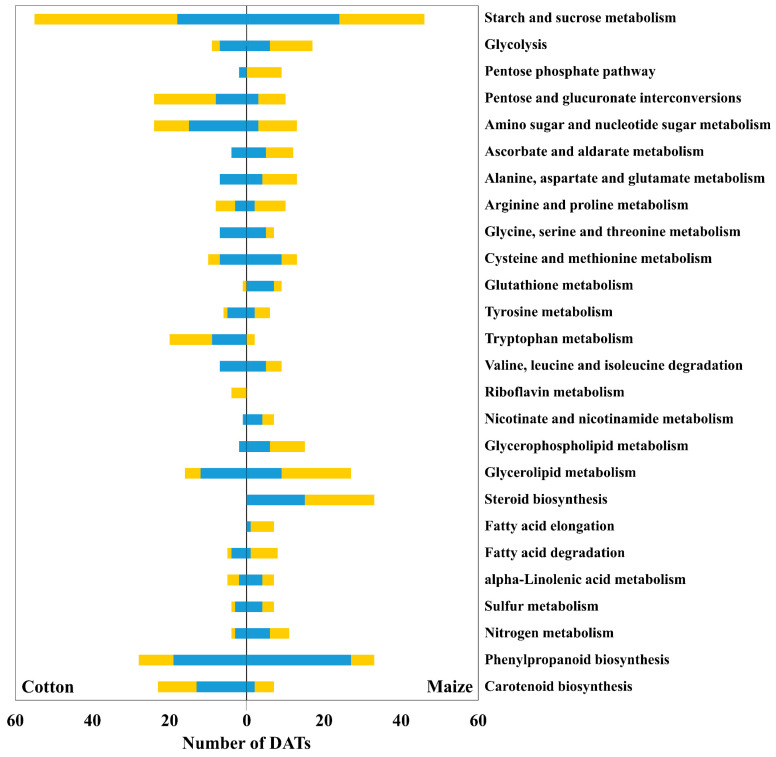
KEGG pathways with top numbers of participating DATs in the cotton and maize primary root elongation zones at 48 h after transplanting to the water stress treatment. The 20 highest ranked pathways in each species (according to the number of participating DATs), together with pathway differences that were distinguished between the species, are presented. Yellow bars indicate DATs with increased abundance; blue bars indicate DATs with decreased abundance. Left panel: cotton; right panel: maize.

DATs at 48 h after water stress imposition were analyzed to determine enriched GO terms. All significant biological process terms in cotton, with their corresponding values in maize, plus 10 additional biological process terms with the highest significances in maize but not represented in cotton, are shown in Figure 2. The GO term enrichment results for all significant biological process, molecular function and cellular component categories terms are summarized in Supplemental Data S1. To determine pathways that were highly involved in the responses to water stress, DATs in each species were mapped to pathways in the KEGG database. The 20 highest ranked pathways in each species (according to the number of participating DATs), together with pathway differences that were distinguished between the species, are presented in Figure 3.

In cotton, 591 DATs increased, and 931 DATs decreased in abundance at 48 h after stress imposition (Table S1). Of these, 87% (90% of positive DATs and 86% of negative DATs) exhibited similar responses at 24 h, indicating a stable transcriptomic response. Of the 1522 total DATs in cotton, 1019 were assigned GO terms and subjected to GO category enrichment analysis that spanned 20 biological processes (Figure 2 and Figure S1; Supplementary Data S1) and 253 were mapped to 40 metabolic pathways (Figure 3; Table S3). In water-stressed maize roots, 886 DATs increased, and 746 DATs decreased in abundance at the 48 h time point (Table S2). Of these, 92% (96% of positive DATs and 86% of negative DATs) exhibited similar responses at 24 h, indicating a stable transcriptomic response as seen for cotton. Of the 1632 total DATs in maize, 1317 were assigned GO terms and subjected to GO category enrichment analysis that spanned 54 biological processes (Figure 2 and Figure S2; Supplementary Data S1) and 277 were mapped to 40 metabolic pathways (Figure 3; Table S3).

The GO category enrichment analysis of the cotton and maize transcriptome responses underscores both the similarities and differences between the primary root elongation zone response to water stress in the two species (Figure 2). It appears that the stress response was more expansive, involving more biological processes, in maize compared with cotton, even though the tissue water status and cellular growth responses were almost identical in the two species [11]. This may be a reflection of the adaptation of cotton to more arid environments [24,25]. It is noteworthy, in this regard, that only the maize primary root elongation zone was enriched for transcripts that are classified as responsive to abscisic acid (ABA), which is an important phytohormone in many responses to abiotic stress [26]. In cotton, the transcriptome responded to water deficit-stress primarily by altering the abundance of transcripts involved in carbohydrate and amino acid metabolism, presumably in association with osmoregulatory responses [27,28,29,30]. The maize transcriptome also responded to water stress by impacting the abundance of transcripts involved in osmoregulatory processes. However, unlike cotton, the maize response also involved transcripts related to ROS metabolism that are commonly associated with water deficit stress and other abiotic stress conditions [31]. The majority of the maize DATs associated with ROS metabolism (Table S4) were cytochrome P450 proteins, which, as discussed later, are likely involved in membrane repair associated with ROS activity. There were also several peroxidase DATs and two catalase DATs that accumulated in response to the water deficit treatment of maize. However, as demonstrated in the previous study by Kang et al. [11], the increases in catalase transcript abundance did not equate to an increase in enzyme activity. This observation supports the results of our metabolic study, which demonstrated that the maize primary root elongation zone experienced significantly increased levels of H_2_O_2_ during the water-stress treatment, whereas this response was not observed in the cotton counterpart [11].

The KEGG pathway analysis and comparison of the water-stress responsive DATs between the root elongation zones of the two species (Figure 3 and Table S3) allow for a closer look at the transcriptomic response in relation to the physiological, metabolomic and enzymatic analyses of these tissues [11]. Overall, the analysis revealed that pathways represented by significant alterations in transcript abundance were largely similar in the two species and consistent with the GO category enrichment analysis. However, steroid biosynthesis and fatty acid elongation pathway DATs are represented in the water-stressed maize root elongation zone but not in the equivalent tissue in cotton, and the riboflavin pathway is represented in the water-stressed cotton DATs but not in maize. In the metabolite profiling of the primary root elongation zones of these two species, the analysis of plant sterols was limited to six metabolites; 3-hydroxy-3-methylglutarate, beta-sitosterol, campesterol, ergosterol, fucosterol, and stigmasterol [11]. In water-stressed cotton roots, only beta-sitosterol and stigmasterol exhibited positive changes in abundance, whereas in the equivalent tissues for maize, beta-sitosterol and ergosterol significantly increased in abundance whilst the remaining three sterols declined significantly. The changes in sterol levels observed in water-stressed cotton roots were not reflected in significant changes in the abundance of transcripts involved in sterol biosynthesis. No cotton DATs could be attributed to this aspect of metabolism (Figure 3), which, therefore, is likely controlled at the physiological and metabolic levels in the cotton root. In the maize root elongation zone, in contrast, 33 DATs were associated with the response of steroid metabolism to water stress (Figure 3 and Table S3), 18 of which increased in abundance (Table S5). The difference in the number of DATs associated with steroid metabolism between the species, zero in cotton and 33 in maize, is suggestive of a physiological and metabolic water stress response in the maize root elongation zone that is lacking in cotton. Seventeen of the DATs that accumulated in maize were transcripts that encode P450 proteins, and the remaining transcript encodes a sterol methyltransferase. As the most common reactions catalyzed by P450 proteins are hydroxylation and desaturation [32], it is possible that water stress initiates the modification, damage, or repair of membrane phytosterols in the maize root elongation zone, but this is speculative. Further understanding of the sterol-related responses to water stress requires a dedicated analysis of sterol metabolism in the root elongation zone for both species.

In the maize primary root, water stress-positive DATs associated with fatty acid elongation primarily encode 3-ketoacyl-CoA synthases (5 of 6), with a single transcript encoding beta-ketoacyl reductase 1 (Table S5). The proteins encoded by these transcripts are components of the endoplasmic reticulum membrane-bound fatty acid elongase complex [33], which synthesizes very-long-chain fatty acids that in leaves are incorporated into cuticular waxes and in roots into suberin [34]. The measurement of suberin levels in maize (or cotton) primary roots in response to water stress was not a feature of our previous metabolic report [11], but a water stress-induced increase in suberin has been reported for maize roots [35]. In addition, ABA, which increases in the elongation zone of water-stressed maize primary roots [14], also promotes suberin biosynthesis and deposition [36]. Changes in suberin levels can have significant effects on the hydraulic conductivity of roots, as discussed in Grünhoffer et al. [35].

The lone KEGG pathway that was populated by stress-responsive DATs only in the cotton primary root elongation zone was the riboflavin pathway, which was exclusively represented by positive changes in the abundance of transcripts encoding purple acid phosphatases (Table S5). These enzymes are commonly associated with phosphate starvation and are secreted into the rhizosphere to scavenge for this element [37]. The vermiculite system used in this study was designed to rely on stored seed reserves to fuel the growth of the seedlings. It is possible that for cotton there are insufficient stores of phosphate to cope with the rigors of a water deficit stress and, thus, the seedling responds by increasing the transcript abundance of purple acid phosphatases. It is worth noting that drought tolerance in cotton is closely linked to soil phosphate levels—the more phosphate in the soil, the more tolerant cotton is to the effects of drought [38,39].

### 3.2. Antioxidative and Sulfur Metabolism

**Figure 4:**
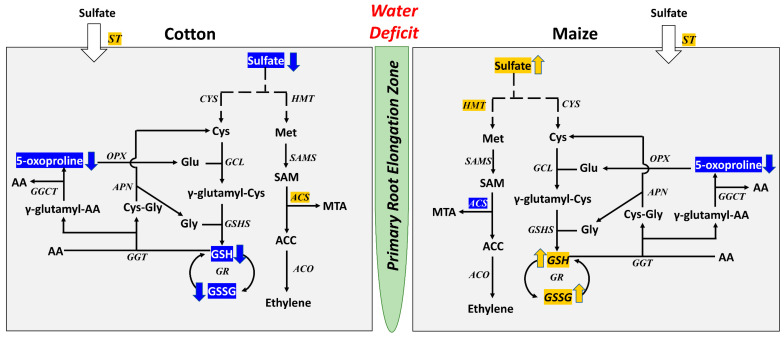
Summary of key changes of encoding transcripts of enzymes and metabolites in the primary root elongation zone of cotton (**left panel**) and maize (**right panel**) seedlings growing under water deficit conditions with a focus on sulfur and glutathione metabolism. Italic fonts represent key enzymes that have at least one encoding transcript increased (highlighted in yellow) or decreased (highlighted in blue) in abundance in water-stressed roots compared with well-watered controls. Arrows adjacent to the metabolites indicate increases (yellow) or decreases (blue) in abundance in water-stressed compared with well-watered roots. Enzymes with black font and no arrow beside the metabolite indicate no significant change. Abbreviations: ST, sulfate transporter; Cys, cysteine; Glu, glutamate; Gly, glycine; GSH, glutathione (reduced); GSSG, glutathione (oxidized); AA, amino acid; Met, methionine; SAM, S-adenosylmethionine; MTA, 5′-methylthioadenosine; ACC, 1-aminocyclopropane-1-carboxylate; CYS, cysteine synthase; GCL, glutamate-cysteine ligase; GSHS, glutathione synthase; GR, glutathione reductase; GGT, gamma-glutamyltranspeptidase; GGCT, gamma-glutamylcyclotransferase; OPX, oxoprolinase; APN, aminopeptidase N; HMT, homocysteine methyltransferase; SAMS, S-adenosylmethionine synthetase; ACS, 1-aminocyclopropane-1-carboxylate synthase; ACO, aminocyclopropane-carboxylate oxidase.

In the preceding metabolic study, alterations in antioxidative and sulfur metabolism, primarily involving differential responses of glutathione levels, were identified as key metabolic differences between the water stress responses of the cotton and maize primary root elongation zones [11]. The seminal observation was that, in cotton, glutathione levels declined during the 48 h water stress treatment, whereas in maize, glutathione levels in water-stressed roots were significantly elevated above the well-watered control levels throughout the experiments. Interestingly, the transcriptome data do not predict or correlate with these observations in either species, as evidenced by the KEGG analysis of DATs (Figure 3, Table S5). In cotton, no DATs that encode any of the key enzymes in the glutathione cycle exhibited a decrease in transcript abundance. This is also the case for the transcriptome response of water-stressed maize roots, which did not include DATs encoding glutathione cycle enzymes, whether representing transcripts that increased or decreased in abundance (Figure 3, Table S5). The water stress-responsive transcriptomes of both species include DATs associated with the glutathione pathway, but all are associated with processes that utilize glutathione rather than contribute to its synthesis or direct conversion between the reduced and oxidized forms. DATs encoding glutathione S-transferases (GST) occurred in the stress-responsive transcriptomes of both species; in cotton, a single GST DAT exhibited an increase in abundance, whereas in maize there were seven GST DATs that all declined in abundance. GSTs catalyze the conjugation of glutathione (in the reduced form, GSH) to a wide range of electrophilic substrates and often function as detoxifying agents to remove toxic lipid peroxidation products and damaged DNA products resulting from oxidative stress [40]. It is possible that an increase in GST activity resulting from an increase in transcript abundance (assuming it is translated) might lead to the decline in glutathione levels observed in the primary root elongation zone of cotton during water stress. Conversely, a decrease in GST activity resulting from the decline in transcript abundance for seven GSTs might lead to an increase in glutathione in water-stressed maize roots, but this seems unlikely. Such a scenario would require greater oxidative stress in the cotton root elongation zone during water stress and a diminished level of oxidative stress in the maize counterpart, requiring greater and lesser protection, respectively. However, this is the opposite of what we observed in our metabolite study [11]. It is more likely that the decline of glutathione in cotton and increase of glutathione in maize primary root elongation zones during water stress are controlled by metabolic regulation of the glutathione cycle and not by changes in the transcripts encoding proteins that are indirectly associated with the pathway (summarized in Figure 4).

In conjunction with the contrasting water stress-induced changes in glutathione metabolism between the elongation zones of cotton and maize primary roots, sulfate levels declined significantly in cotton and increased significantly in the maize root tissues [11]. The transcriptomes for each species exhibited DATs encoding sulfate transporter proteins that have the potential to mediate these changes. In cotton, two sulfate transporter DATs decreased in abundance and one increased in abundance and, if the transcript abundance reflects the associated protein abundance, the net effect would be a reduction in sulfate uptake as suggested by the metabolomic analysis. It could also be possible that the decrease in sulfate levels in the root tissues activates the increase in abundance of one of the sulfate transporter genes, but it was insufficient to negate the loss of sulfate at 48 h after water stress imposition. In the elongation zone of water-stressed maize roots, two sulfate transporter DATs increased in abundance and two decreased, in association with the net increase in sulfate level. Without detailed genetic and biochemical studies it is difficult to determine if the changes in transcript abundance in the root tissues of either species were associated with changes in the genetic control of sulfate uptake, or whether physiological changes in the root elongation zones during exposure to water stress resulted in the contrasting effects on sulfate levels.

### 3.3. Ortholog Analysis

**Figure 5:**
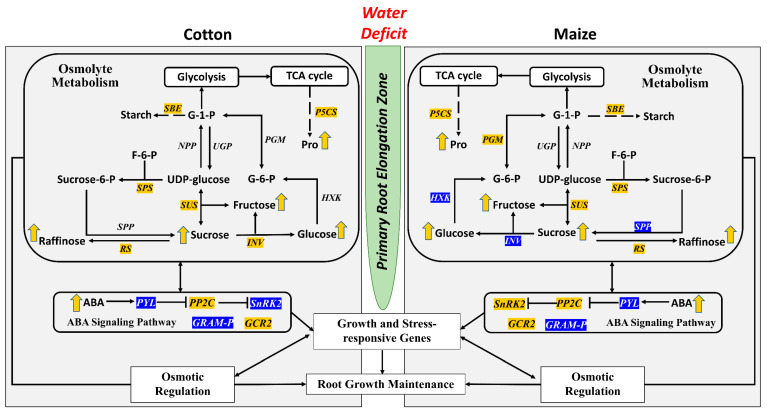
Summary of key changes of encoding transcripts of enzymes and metabolites in the primary root elongation zone of cotton (**left panel**) and maize (**right panel**) seedlings growing under water deficit conditions with a focus on osmolyte metabolism and the ABA signaling pathway. Italic fonts represent key enzymes that have at least one encoding transcript increased (highlighted in yellow) or decreased (highlighted in blue) in abundance in water-stressed roots compared with well-watered controls. Arrows adjacent to the metabolites indicate increases (yellow) or decreases (blue) in abundance in water-stressed compared with well-watered roots. Enzymes with black font and no arrow beside the metabolite indicate no significant change. Abbreviations: G-1-P, glucose-1-phosphate; F-6-P, fructose-6-phosphate; Sucrose-6-P, sucrose-6-phosphate; G-6-P, glucose-6-phosphate; Pro, proline; ABA, abscisic acid; SBE, starch branching enzyme; NPP, nucleotide diphosphatase; UGP, UDP glucose pyrophosphorylase; PGM, phosphoglucomutase; SUS, sucrose synthase; SPS, sucrose-phosphate synthase; SPP, sucrose-phosphate phosphatase; INV, invertase; HXK, hexokinase; RS, raffinose synthase; P5CS, delta 1-pyrroline-5-carboxylate synthase; PYL, PYR1-like; PP2C, protein phosphatase 2C; SnRK2, SNF1-related protein kinase; GRAM-P, GRAM domain-containing ABA-responsive protein; GCR2, G protein coupled receptor 2.

To compare the water stress-responsive transcriptomes of the cotton and maize primary root elongation zones more directly and to better link them to the previous metabolite data, the DATs were subjected to an ortholog analysis to determine if the genes they represent are orthologous between the two species. Orthogroups were generated utilizing the (*Gossypium hirsutum*) genome (NAU-NBT v1.1) and the maize (*Zea mays*) genome (AGPv4) with a specific assembly for cv. FR697 [17], combined with genome resources from 27 other plant species (see Section 2). The combined 3154 DATs were cataloged into 1223 of the generated orthogroups, of which only 173 (14%) contained DATs from both species (Table S6). This finding implies that much of the primary root elongation zone responses of the two species to water stress is phylogenetically unrelated and could be considered at least lineage restricted. This result also indicates that 14% of the response utilizes orthologous genes and could be considered conserved between cotton and maize, and, thus, to predate the dicot/monocot divergence. To examine further which aspects of the water stress responses of the two species are conserved, or phylogenetically linked, DATs that have one-to-one matched orthologs were identified and filtered into their corresponding GO term categories, resulting in 501 DATs, 286 for cotton and 215 for maize, spanning 162 orthogroups (Table S7). The majority of the one-to-one orthologs fall into three main metabolic categories; ABA biosynthesis and receptors, amino acid metabolism, and carbohydrate metabolism (summarized in Figure 5). In addition, it is of note that sulfate transporter DATs, although in one case with contrasting changes in abundance, are also represented by one-to-one ortholog matches indicating the conserved nature of this aspect of the water stress response.

ABA exerts hormonal control to coordinate the physiological and metabolic responses of plants to abiotic stresses, including responses to water stress and desiccation [41]. ABA biosynthesis and its associated signaling pathway has origins deep in the land plant phylogeny and is genetically highly conserved [42,43]. ABA is synthesized both in plastids and the cytosol from isopentenyl diphosphate via a complex biosynthetic pathway, the rate limiting step of which involves the enzyme nine-cis-epoxycarotenoid dioxygenase (NCED) that utilizes violaxanthin and neoxanthin as substrates [44]. The ABA signaling pathway is regulated by the ABA-Pyrabactin Resistance1 (PYR1)/PYR1-Like/Regulatory Component of ABA Receptor (PYR/PYL/RCAR)/ Protein Phosphatase 2C (PP2C)–SNF1-Related Protein Kinase 2 (SnRK2) module [26]. ABA accumulates in the primary root elongation zone of maize and cotton under water-stressed conditions similar to those imposed in this study [14,45], and this is reflected in the observation that NCED transcripts accumulated in the transcriptomes of both species (Tables S1 and S2). Not only were DATs for NCED increased in abundance for both species; they also derived from directly orthologous genes (Table S7). The major component of the ABA signaling pathway that appeared to be regulated by water stress in the root elongation zone for both cotton and maize was the PP2C phosphatase, which comprised a significant number of the positive DATs for each transcriptome. As observed for the NCED DATs, the PP2C DATs arose from directly orthologous genes for cotton and maize, reflecting the conserved nature of the response (Table S7). PP2C phosphatases have a dual role in ABA signaling—in the absence of ABA they bind to the SnRK2 kinase and inhibit its activity, thus preventing its phosphorylation of the downstream effector, whereas in the presence of ABA, PP2Cs bind to ABA receptors to capture ABA [46]. High levels of PP2Cs can also act as a negative feedback mechanism that desensitizes plants to high ABA levels [47]. Without more detailed biochemical and genetic data, it is difficult to explain the role of an increase in PP2C transcripts in the primary root responses to water stress, but it is interesting to speculate that the high level of PP2C might be required to control the balance between ABA-mediated maintenance of growth in the apical 3 mm of the root tips during water stress [48] and the growth inhibitory aspects that an increase in ABA can induce [49,50].

Osmotic adjustment is a fundamental response to low water potentials that lessens tissue water loss by reducing cellular osmotic potential [30] and occurs in cells from algae to angiosperms. Osmotic adjustment is an important aspect of root growth maintenance under water stress [27,28,29]. Accumulations of osmolytes, including the two primary compounds sucrose and proline, were identified in the primary root elongation zones of both cotton and maize (Figure 5), consistent with roles in maintaining hydration and viability as well as in turgor maintenance for cell expansion [11]. DATs encoding sucrose phosphate synthase (SPS) and delta1-pyrroline-5-carboxylate synthase (P5CS), key enzymes in sucrose and proline biosynthesis, respectively, as well as sucrose synthase (SUS), an enzyme that can catabolize or synthesize sucrose in sink tissues such as roots [51], all accumulated in the stressed roots of both species (Tables S1 and S2). The conserved nature of this aspect of the water stress response is highlighted by the fact that DATs for each of the three key enzymes, SPS, P5CS, and SUS, are represented by one-to-one orthologs for the two species (Table S7).

## 4. Conclusions

Soil water deficit leading to tissue dehydration provides a powerful selection pressure to evolve tolerance mechanisms because water limitation is such an existential threat. The transcriptome responses of the primary root elongation zones of cotton and maize to equivalent levels of tissue water stress support our previous physiological and metabolic findings that both conserved and species-specific responses are activated. The conserved nature of elevated transcript abundances for genes involved in ABA biosynthesis and signaling, as well as key enzymes that enable osmotic adjustment, point towards the adaptive nature of the response of these two processes in water-stressed roots. However, ortholog analysis revealed that 86% of the transcriptome response of the primary root elongation zone to water stress was phylogenetically unrelated between cotton and maize. In particular, the antioxidant response in water-stressed roots, at least with regard to glutathione metabolism and anti-oxidative enzymes, does not have such a transcript abundance adaptive signature that could be uncovered by the ortholog analysis. The differential responses of the glutathione and sulfur metabolic pathways we previously reported for cotton and maize were not evident in the transcriptomic responses, implying that the primary root elongation zone of both species copes with water stress-induced oxidative stress by responding metabolically, more so for maize than cotton, and by providing ample antioxidant enzyme activities within the root tissues. This finding indicates that the antioxidant response in both species results from metabolic acclimation to the water deficit stress, and thus represents an example of water stress-related metabolic plasticity.

## Data Availability

Data are contained within the article and Supplementary Materials. RNA-seq data are available from the GEO database (Accession number: GSE221296).

## Supplementary Materials

The following supporting information can be downloaded at: https://www.mdpi.com/article/10.3390/antiox12020287/s1, Figure S1: Pipeline of procedures for comparative transcriptomic and orthologous analyses to identify key water stress-responsive DATs and orthologs in the cotton and maize primary root elongation zones; Table S1: Ratios of transcript abundance of DATs that significantly changed in the elongation zone of cotton primary roots at 24 h and 48 h after water stress imposition compared with well-watered controls; Table S2: Ratios of transcript abundance of DATs that significantly changed in the elongation zone of maize primary roots at 24 h and 48 h after water stress imposition compared with well-watered controls; Table S3: Numbers of increased and decreased DATs assigned to KEGG pathways in cotton and maize primary root elongation zones in response to water stress; Table S4: Ratios of transcript abundance of DATs related to ROS metabolism that significantly changed in the elongation zone of maize primary roots at 24 h and 48 h after water stress imposition compared with well-watered controls; Table S5: DATs in KEGG pathways with distinguished differences between cotton and maize primary roots in response to water stress with their KEGG Orthology IDs; Table S6: Ortholog matches that contain DATs in cotton and maize primary roots in response to water stress identified with both ortholog analysis methods with the ortholog group IDs; Table S7: Ortholog group IDs and ratios of transcript abundance for orthologs that were DATs in both cotton and maize primary roots in response to water stress identified with different methods; Supplementary Data S1: Significant GO terms in the elongation zones of cotton and maize primary roots in response to water stress.
