# Supplementary material for: Comparative Transcriptomics Reveal Metabolic Rather than Genetic Control of Divergent Antioxidant Metabolism in the Primary Root Elongation Zone of Water-Stressed Cotton and Maize
Source: Antioxidants (Basel). 2023 Jan 27;12(2):287. doi: 10.3390/antiox12020287 (PMC9952253; doi:10.3390/antiox12020287)

Figure S1. Pipeline of procedures for comparative transcriptomic and orthologous analyses to identify key water stress-responsive DATs and orthologs in the cotton and maize primary root elongation zones. WW, well-watered; WS, water-stressed.

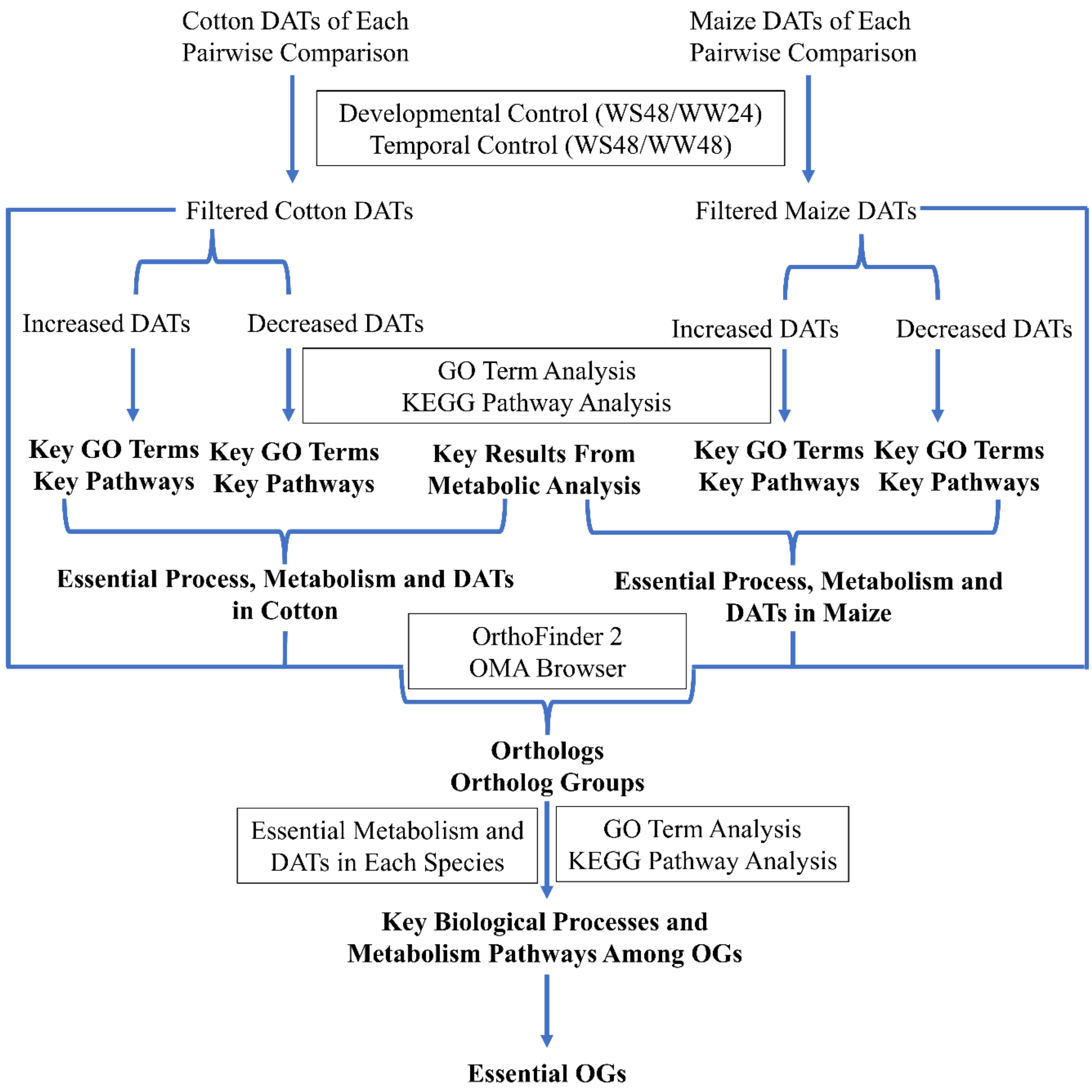

Supplement: Supplementary file 1 [file antioxidants-12-00287-s001.zip › Figure S1.pdf]
